# Genotype–phenotype correlations in pediatric CAPS with predominantly low-penetrance NLRP3 variants among Turkish patients in Germany and Turkey: beyond borders

**DOI:** 10.1186/s12969-026-01214-7

**Published:** 2026-04-11

**Authors:** Yagmur Bayindir, Özlem Satirer, Veysel Cam, Seyma Turkmen, Betul Sozeri, Jasmin B. Kuemmerle-Deschner, Seza Ozen

**Affiliations:** 1 Department of Pediatric Rheumatology, Faculty of Medicine, Hacettepe University, Ankara, Turkey; 2 Paediatric Rheumatology, Department of Paediatrics and Autoinflammation Reference Center Tuebingen (arcT), University Hospital Tuebingen,Member of ERN-RITA, Tuebingen, Germany; 3 Department of Pediatric Rheumatology, Umraniye Training and Research Hospital, Health Sciences University, Istanbul, Turkey; 4 Department of Paediatrics, University Children’s Hospital Tuebingen, Hoppe-Seyler-Strasse 1, Tuebingen, 72076 Germany

**Keywords:** Cryopyrin-associated periodic syndrome, Phenotype and genotype, Geographic, VUS

## Abstract

**Background:**

The phenotypic heterogeneity and variable disease course of CAPS cannot be explained solely by *NLRP3* mutations, suggesting additional environmental or modifying factors. This study aimed to characterize the phenotypic and genotypic features of Turkish-origin pediatric CAPS patients residing in Turkey (Turkish cohort) and Germany (German cohort).

**Methods:**

This multicenter, retrospective study included Turkish-origin pediatric patients meeting CAPS criteria from centers in Germany and Turkey. Demographic, genetic, clinical, and treatment data were collected, and disease activity was assessed using PGA and PPGA.

**Results:**

The study included 51 Turkish-origin pediatric CAPS patients (living in Turkey: 23; Germany: 28) with a median age of disease onset at 1 year and diagnosis at 4 years. The Turkish cohort had more pathogenic mutations and more severe phenotypes, while the German cohort predominantly carried VUS/Q703K variants with a mild phenotype. Diagnostic delay and attack duration were longer in the Turkish cohort; urticarial rash, fatigue, thoracic pain, fever, and aphthous ulcers were more frequent, whereas diarrhea, lymphadenopathy, and infection-triggered attacks predominated in the German cohort. Baseline disease activity was higher in the Turkish cohort, and most patients achieved remission at last visit under therapy—mainly above-standard doses of Canakinumab—whereas over half of the German cohort reached remission without treatment. **VUS and Q703K subgroup**: Among patients with similar genotypes (VUS/Q703K), the Turkish cohort presented with older age at diagnosis, longer diagnostic delay, more moderate phenotypes, and higher baseline disease activity, whereas the German cohort exhibited milder phenotypes, infection-triggered attacks, and gastrointestinal involvement. The Turkish cohort received Canakinumab or Anakinra more frequently, often with dose adjustments and above-standard doses, achieving complete remission frequently under therapy at last visit, while the German cohort more often reached remission without treatment. Non-remission and partial remission were observed exclusively in the Turkish cohort.

**Conclusion:**

These findings suggest that Turkish-origin pediatric CAPS patients living in Turkey, including those with similar VUS/Q703K genotypes, may experience higher disease activity and greater treatment dependence than their German counterparts. However, as most patients in our cohort carried low-penetrance VUS variants, these observations are not directly generalizable to individuals with clearly pathogenic NLRP3 genotypes. Overall, this supports the notion that factors beyond genetics, potentially including environmental or modifying influences, contribute to CAPS severity and warrant further investigation.

**Supplementary Information:**

The online version contains supplementary material available at 10.1186/s12969-026-01214-7.

## Background

Cryopyrin-Associated Periodic Syndromes (CAPS) are rare monogenic autoinflammatory diseases with substantial individual and societal burden [1]. They result from gain-of-function mutations in the *NLRP3* gene encoding cryopyrin [2, 3], leading to constitutive *NLRP3* inflammasome activation and excessive secretion of interleukin-1β (IL-1β) [4, 5]. IL-1 inhibition effectively suppresses inflammation, controls disease activity, prevents organ damage, and improves quality of life [1, 5, 6].

CAPS encompass a clinical spectrum ranging from mild Familial Cold Autoinflammatory Syndrome (FCAS) to moderate Muckle–Wells Syndrome (MWS) and severe Chronic Infantile Neurologic Cutaneous Articular Syndrome/Neonatal-Onset Multisystem Inflammatory Disease (CINCA/NOMID). While IL-1 blockade is universally recommended, treatment requirements differ considerably across the spectrum [1, 7–9].

The phenotypic expression of CAPS is highly variable even among individuals carrying identical *NLRP3* variants, suggesting that disease severity is not determined by genotype alone. In particular, the pathogenic contribution of variants of uncertain significance (VUS) remains controversial, as these variants may manifest with either classical CAPS or milder, PFAPA- or SURF-like phenotypes [10]. This variability implies that non-genetic modifiers—such as environmental exposures, climate, infection patterns, and healthcare access—may significantly shape disease expression.

Despite accumulating clinical experience, systematic analyses comparing the same ethnic background under different environmental contexts are lacking. Turkish-origin patients, residing in Turkey and Germany, offer a unique opportunity to disentangle the influence of environment from genetic background.

Therefore, the aim of the study was (1) to characterize the phenotypic and genotypic characteristics of consecutively enrolled Turkish-origin pediatric CAPS patients residing in Turkey and Germany, and (2) to compare clinical features and treatment responses across the general cohort and genotype-matched subgroups, focusing on VUS carriers, particularly the Q703K (HGVS: NLRP3:c.2107 C > A p.(Q705K)) variant.

## Methods

A multicenter study was conducted involving pediatric CAPS patients of Turkish descent living in Germany (German cohort) and Turkey (Turkish cohort). Patients were included based on the following criteria: (1) age at diagnosis under 18 years and fulfillment of CAPS classification and/or diagnostic criteria [11, 12] and (2) longitudinal follow-up with at least three visits at the respective centers. Patients were excluded if they (1) had not lived in the country of the cohort since birth /had been relocated later, or (2) had additional comorbidities or medications affecting outcomes. Clinical data were retrospectively collected between January and July 2024 using a standardized case report form. The data sources included patient files and research databases. The study protocol was approved by the institutional review board at each recruiting center.

### Data

Patient- and disease-related information was collected, including gender, age at disease onset, age at diagnosis, and age at the start of therapy. Clinical symptoms associated with CAPS, the average attack duration and frequency at both the initial and most recent visits, as well as the CAPS phenotype (mild-FCAS, moderate-MWS, severe-CINCA/NOMID), were recorded. In addition, *NLRP3* variants, including the American College of Medical Genetics and Genomics (ACMG) classification, were captured. All patients underwent a comprehensive autoinflammatory gene panel analysis, including NLRP3, MEFV, MVK, and NLRC4. Damage findings, including hearing loss and amyloidosis, were recorded in both cohorts.

### Monitoring instruments

Inflammatory biomarkers were measured, and disease activity was clinically assessed using the Physician Global Assessment (PGA) and Patient/Parent Global Assessment (PPGA). The PGA and PPGA recorded disease activity on a 10 cm visual analog scale (VAS), where 0 represented no disease activity and 10 represented maximal disease activity.

### Remission definitions

- **Complete remission** was defined as PGA ≤ 2, with CRP ≤ 0.5 mg/dl and/or SAA ≤ 10 mg/L.
- **Partial remission** was defined as PGA > 2 and ≤ 5, with CRP > 0.5 mg/dl but ≤ 3 mg/dl and/or SAA > 10 mg/L but ≤ 30 mg/dl.
- **Non-remission** was defined as PGA > 5, with CRP > 3 mg/dl and/or SAA > 30 mg/dl [13, 14].

### Treatments

In both the German and Turkish cohorts, all patients were initially started on standard doses of treatment. Canakinumab was administered as an initial regimen at a dose of 150 mg for adults or 2 mg/kg for children under 40 kg, delivered subcutaneously every 8 weeks. Anakinra was prescribed at a **standard dose (SD)** of 1–2 mg/kg/day [1]. For colchicine, the recommended starting dose was used by age: ≤ 0.5 mg/day for children under 5 years, 0.5–1.0 mg/day for children aged 5–10 years, and 1.0–1.5 mg/day for children over 10 years of age [15]. The following terminology was applied to describe dosing regimens in this study:

- **SD**: Standard dose
- **> SD**: Above standard dose

### Visitation and examinations

- **First Visit**: The initial visit of patients at the center.
- **Follow-up Visits**: Follow-up examinations were conducted at intervals of 3–6 months after diagnosis and the commencement of therapy.
- **Last Visit**: The final visit of patients at the center during the study period.

### Analysis

Descriptive statistics summarized patient characteristics, with categorical variables as frequencies/percentages and continuous variables as mean ± SD or median (range), based on data distribution. Normality was assessed using Shapiro-Wilk and Kolmogorov-Smirnov tests. Group comparisons employed t-tests or Mann-Whitney U/Wilcoxon tests as appropriate. Analyses were performed using SPSS 28.0.1.1 and STAT software, with significance set at *p* < 0.05.

## Results

This study included 51 pediatric CAPS patients of Turkish origin (25 females, 26 males), with 23 living in Turkey and 28 in Germany. The median age at disease onset was 1 year, and at diagnosis it was 4 years, with a median diagnostic delay of 2 years. Patients were followed for a median of 6 years, and the average attack duration was 4 days.

Genetic analysis revealed that 76% of patients had variants of uncertain significance (VUS), 64% carried the Q703K mutation, 16% had pathogenic or likely pathogenic mutations, 6% had no identified mutations, and 2% had benign mutations. Clinically, 77% of patients had moderate Muckle-Wells Syndrome (MWS), 14% had mild Familial Cold Autoinflammatory Syndrome (FCAS), and 9% had severe Chronic Infantile Neurological, Cutaneous, and Articular Syndrome/Neonatal Onset Multisystem Inflammatory Disease (CINCA/NOMID).

The most common clinical manifestations were inflammatory flares (87%), fatigue (81%), urticarial rash (77%), arthralgia (73%), and abdominal pain (60%). Other symptoms included oral aphthae (58%), headache (48%), lymphadenopathy (48%), conjunctivitis (33%), diarrhea (31%), arthritis (29%), and stress (15%). Less common complications were frontal bossing (6%), aseptic meningitis (8%), hearing loss (2%), and amyloidosis (2%). Triggers for attacks included infections (44%), cold exposure (23%), and seasonal changes (23%).

At baseline, both the median PGA and PPGA scores were 7. The median baseline CRP level was 3.9 mg/dL. At the final follow-up, the median PGA and PPGA scores were 0, and the median CRP level was 0.1 mg/dl. The median attack frequency in the last year was 0. Regarding treatment, 72% of patients initially received colchicine, while 14% were started on canakinumab and 14% on anakinra (Table 1).

**Table 1:** Baseline characteristics of 51 consecutive pediatric CAPS patients of Turkish Origin living in Germany and Turkey

Demographics	
Total Patients	51
Gender (Female/Male)	25/26
Age at Disease Onset in years, median (range)	1 (0–13)
Age at Diagnosis in years, median (range)	4 (1–18)
Diagnostic Delay in years, median (range)	2 (0–13)
Follow-Up Duration in years, median (range)	6 (1–17)
Attack Length in days, median (range)	4 (2–12)
Genetic Findings *	
Pathogenic/Likely Pathogenic Mutations, n (%)	8 (16)
Variants of Uncertain Significance, n (%)	39 (76)
No Identified Mutations, n (%)	3 (6)
Benign Mutations, n (%)	1 (2)
**Phenotype**	
Moderate-Muckle-Wells Syndrome n (%)	40 (77)
Mild-Familial Cold Autoinflammatory Syndrome n (%)	7 (14)
Severe-CINCA/NOMID n (%)	4 (9)
**Baseline Measures**	
Initial PGA, median (range)	7 (2–10)
Initial PPGA, median (range)	7 (2–10)
Initial CRP mg/dl, median (range)	3.9 (0.3–19.2)
**Last Follow-Up Measures**	
PGA, median (range)	0 (0–6)
PPGA, median (range)	0 (0–5)
CRP mg/dl, median (range)	0.1 (0–0.9)
Attack Frequency (last year), median (range)	0 (0–12)
**Initial Treatment**	
Colchicine: n (%)	37 (72)
Canakinumab: n (%)	7 (14)
Anakinra: n (%)	7 (14)

* Pathogenic/likely pathogenic (P/LP) NLRP3 variants (*n* = 8; A441V, D303G, R554) and variants of uncertain significance (VUS, *n* = 39;, Q703K, T198M), plus one benign variant (P340P), are listed. Numbers indicate patients carrying each variant

### Comparison of Turkish and German cohorts

#### Genetic, phenotypic, and clinical characteristics

Among the Turkish cohort (*n* = 23), 30% carried pathogenic or likely pathogenic (P/LP) mutations compared to 3.6% in the German cohort (*n* = 28). Conversely, variants of uncertain significance (VUS) were more frequent in the German cohort (96.4% vs. 70%). The overall difference in mutation type distribution was statistically significant (*p* = 0.009). Phenotypic distribution also differed significantly between cohorts (*p* = 0.023): FCAS occurred exclusively in the German cohort (25%), moderate MWS predominated in the Turkish cohort (87% vs. 71.4%), and severe CINCA/NOMID phenotype was higher in the Turkish cohort (13% vs. 3.6%).

Diagnostic delay was longer in the Turkish cohort (4 vs. 1 year; *p* = 0.004) and initial attack duration was longer (5 vs. 3 days; *p* < 0.001) (Table 2). Clinical symptoms differed: urticarial rash, fatigue, thoracic pain, fever, and aphthous ulcers were more common in the Turkish cohort; diarrhea and lymphadenopathy in the German cohort. Infection-triggered attacks were more frequent in the German cohort (75% vs. 8.7%; *p* < 0.001), while cold/seasonality triggers were higher in the Turkish cohort (39.1% vs. 10.7%; *p* = 0.017). Family history of CAPS was reported more in the German cohort (64.3% vs. 21.7%; *p* = 0.002) (Table 3).

#### Initial and follow-up disease activity assessments

The Turkish cohort showed higher initial CRP levels (median 4 vs. 2.9 mg/dl; p < 0.001) and slightly elevated initial PGA (median 7 vs. 6; p = 0.091) and PPGA scores (median 8 vs. 7; p = 0.153) compared to the German cohort. At last follow-up, PGA and PPGA remained higher in the Turkish cohort (median 1 vs. 0; p = 0.001 and p = 0.046). No significant differences were found in last CRP levels (p = 0.214) or attack frequency (p = 0.811) (Table 2).

#### Treatment approaches, treatment doses and remission status at last visit

57% of patients in the German cohort were untreated, while all patients in the Turkish cohort received treatment (p < 0.001). Canakinumab was the most commonly used therapy in the Turkish cohort (78%) compared to 10% in the German cohort. Colchicine use was higher in the German cohort (28% vs. 8%), and Anakinra was rarely administered in both cohorts, with higher use in the Turkish cohort (13% vs. 3%; p < 0.001) (Fig. 1a). At the last visit, 57% of the German cohort were off medication, whereas all patients in the Turkish cohort were receiving treatment. Moreover, higher-than-standard dosing was more common in the Turkish cohort (56% vs. 10%, p < 0.001). (Fig. 1b). At the last visit, disease activity remained higher in the Turkish cohort. Active disease was present in 26% of the Turkish cohort but in none of the German cohort (p < 0.001). Complete remission without therapy was achieved in 53% of the German cohort, whereas no remission without treatment was observed in the Turkish cohort. In contrast, complete remission with therapy was noted in 65% and partial remission in 8% of the Turkish cohort, compared to 46% and 0% in the German cohort, respectively (Fig. 1c).

**Table 2:** Comparison of genetic, phenotypic, and disease activity assessments between the Turkish and German cohorts

Parameter	Turkish Cohort (*n* = 23)	German Cohort (*n* = 28)	*p*-value (two-sided)
Age at Diagnosis (years), median (range)	13 (5–23)	12.5 (8–21)	0.987
Disease Onset Age (years), median (range)	1 (0–13)	2 (1–12)	0.318
Time to Diagnosis (years), median (range)	4 (0–13)	1 (0–8)	**0.004**
**Mutation Type**			**0.009**
P/LP Mutations, n (%)	7 (30%)	1 (3.6%)	
VUS / Other Mutations, n (%)	16 (70%)	27 (96.4%)	
**Phenotype**			**0.023**
Mild-FCAS, n (%)	0 (0%)	7 (25%)	
Moderate-MWS, n (%)	20 (87%)	20 (71.4%)	
Severe-CINCA, n (%)	3 (13%)	1 (3.6%)	
**Initial and Follow-Up Assessments**			
Initial Attack Length (days), median (range)	5 (2–12)	3 (2–5)	**< 0.001**
Initial PGA, median (range)	7 (2–10)	6 (3–9)	0.091
Initial PPGA, median (range)	8 (2–10)	7 (4–9)	0.153
Initial CRP (mg/dl), median (range)	4 (1.2–19.2)	2.9 (0.3–18.2)	**< 0.001**
Last PGA, median (range)	1 (0–6)	0 (0–2)	**0.001**
Last PPGA, median (range)	1 (0–5)	0 (0–2)	**0.046**
Last CRP (mg/dl), median (range)	0.2 (0.02–0.96)	0.12 (0–0.57)	0.214
Attack Frequency (last year), median (range)	0 (0–12)	0 (0–5)	0.811

**Abbreviations**: P/LP, Pathogenic/Likely Pathogenic; VUS, Variant of Uncertain Significance; FCAS, Familial Cold Autoinflammatory Syndrome; MWS, Muckle–Wells Syndrome; CINCA, Chronic Infantile Neurological Cutaneous and Articular Syndrome; PGA, Physician Global Assessment; PPGA, Patient/Parent Global Assessment; CRP, C-Reactive Protein. **Notes**: p-values were calculated using Pearson’s Chi-square or Mann–Whitney U tests, as appropriate. Ranges indicate minimum and maximum values; all medians are presented as *median (min–max)*

**Table 3:** Comparison of clinical symptoms between the Turkish and German cohorts

Parameter	Turkish Cohort *n* (%)	German Cohort *n* (%)	*p*-value
Headache	11 (47.8)	14 (50.0)	0.877
Urticarial Rash	22 (95.7)	18 (64.3)	**0.007**
Hearing Loss	0 (0.0)	1 (3.6)	0.360
Fatigue	22 (95.7)	20 (71.4)	**0.024**
Amyloidosis	1 (4.3)	0 (0.0)	0.265
Aseptic Meningitis	3 (13.0)	1 (3.6)	0.211
Arthralgia	17 (73.9)	21 (75.0)	0.929
Arthritis	9 (39.1)	6 (21.4)	0.167
Diarrhea	3 (13.0)	13 (46.4)	**0.011**
Vomiting	7 (30.4)	3 (10.7)	0.078
Abdominal Pain	12 (52.2)	19 (67.9)	0.254
Thoracic Pain	4 (17.4)	0 (0.0)	**0.022**
Frontal Bossing	2 (8.7)	1 (3.6)	0.439
Lymphadenopathy	7 (30.4)	18 (64.3)	**0.016**
Papilledema	1 (4.3)	1 (3.6)	0.887
Conjunctivitis	7 (30.4)	10 (35.7)	0.691
Fever	23 (100.0)	22 (78.6)	**0.018**
Aphthous Ulcers	9 (39.1)	21 (75.0)	**0.010**
HSM	2 (8.7)	0 (0.0)	0.111
Family History	5 (21.7)	18 (64.3)	**0.002**
Stress-triggered attacks	4 (17.4)	4 (14.3)	0.762
Infection-triggered attacks	2 (8.7)	21 (75.0)	**< 0.001**
Cold-triggered attacks	9 (39.1)	3 (10.7)	**0.017**
Seasonality-triggered attacks	9 (39.1)	3 (10.7)	**0.017**

Abbreviations and Notes: p-value: Pearson Chi-Square test for categorical variables. HSM: Hepatosplenomegaly. Percentages are rounded to one decimal place. The Total column refers to the sum of participants from Turkey and Germany (*n* = 51)

**Fig. 1a:**
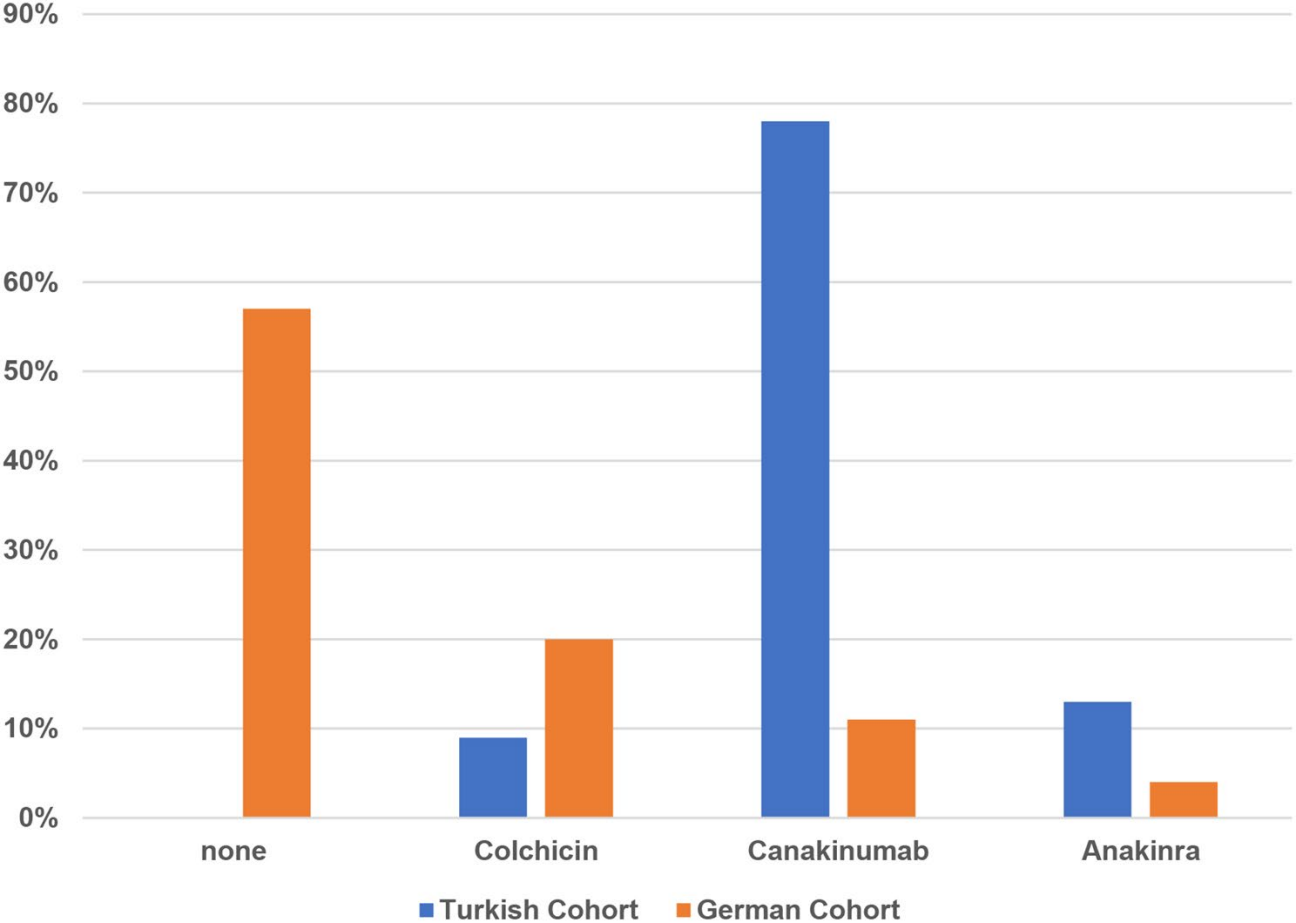
Treatment approaches. Legend: In the Turkish cohort, Canakinumab was used in 78%, Colchicine in 8%, and Anakinra in 13%, with no untreated patients. In the German cohort, 57% were untreated, while Canakinumab, Colchicine, and Anakinra were used in 10%, 28%, and 3%, respectively (*p* < 0.001)

**Fig. 1b:**
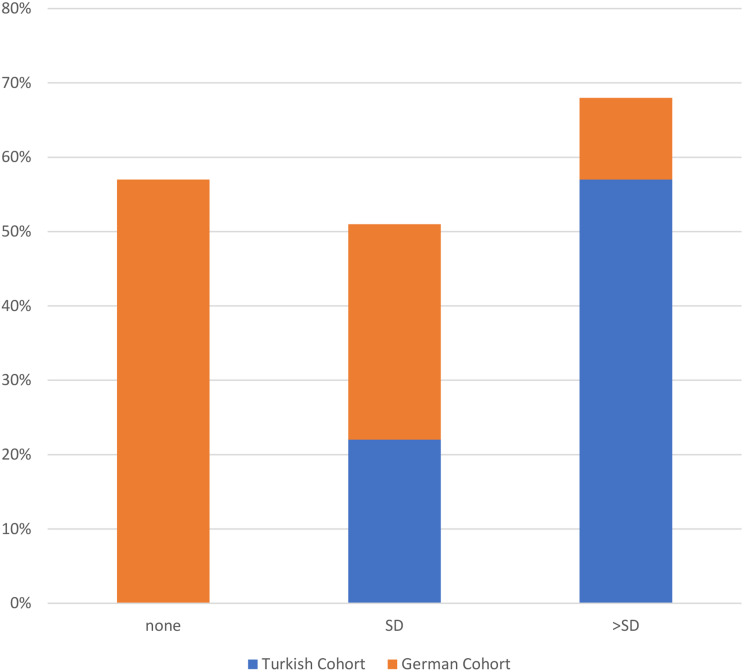
Treatment doses. Legend: Comparison of therapy doses at the last visit in the Turkish (*n* = 23) and German (*n* = 28) cohorts. Doses are categorized as no treatment (none), standard dose (SD), or above the standard dose (> SD). SD: standard dose. Statistical measures include Pearson correlation (*r* = -0.577, SE = 0.094, t = -4.940, *p* < 0.001) and Spearman correlation (-0.586, SE = 0.100, t = -5.062, *p* < 0.001)

**Fig. 1c:**
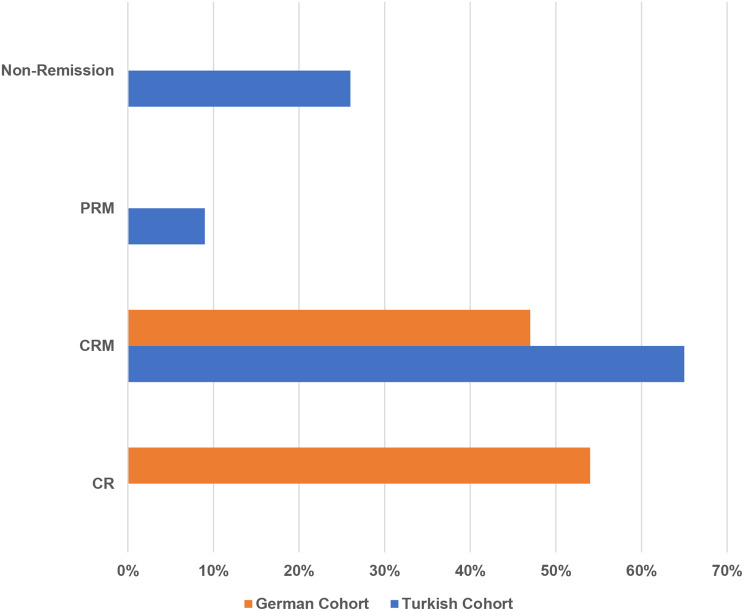
Remission status. Legend: At the last visit, 65% of the Turkish cohort were in CRM, compared to 46% in the German cohort. In contrast, 53% of the German cohort achieved CR, while none of the Turkish cohort reached this status. Non-remission was observed in 26% of the Turkish cohort, but none in the German cohort. PRM was seen in 8% of the Turkish cohort. Chi-square test results: *p* < 0.001. Abbreviations: CRM: Complete remission with therapy, CR: Complete remission without therapy, PRM: Partial remission with therapy

### Comparison of Turkish and German cohorts with VUS mutations

#### Phenotypic and clinical symptoms

Among 39 individuals with VUS mutations, the Turkish cohort had a higher median age at diagnosis (7.5 vs. 3 years, *p* = 0.011) and longer diagnostic delay (4 vs. 1 year, *p* = 0.002). The Turkish cohort exclusively exhibited moderate-MWS, whereas 25% of the German cohort showed mild-FCAS (*p* = 0.016) (Table 4). The Turkish cohort more frequently presented with urticarial rash, arthritis, thoracic pain, frontal bossing, and fever, while diarrhea, lymphadenopathy, aphthous ulcers, and infection-triggered attacks were more common in the German cohort (Table 5). Among 34 individuals carrying the Q703K VUS mutation, findings were consistent: the Turkish cohort had a higher median age at diagnosis (8.7 vs. 3 years, *p* = 0.02) and longer diagnostic delay (6 vs. 1 year, *p* = 0.003), all exhibiting moderate-MWS (25% of the German cohort mild-FCAS, *p* = 0.05) (Supplementary 1). The Turkish cohort showed higher prevalence of urticarial rash and fatigue, whereas lymphadenopathy, aphthous ulcers, and infection-triggered attacks predominated in the German cohort (Supplementary Table 2).

#### Initial and follow-up disease activity assessments

Initial attack length was longer in the Turkish VUS cohort (5 vs. 3 days, *p* = 0.006), with higher initial PGA scores (8 vs. 6, *p* = 0.016) and CRP levels (5.2 vs. 2.9 mg/dl, *p* = 0.002). Last PGA scores remained significantly higher in the Turkish VUS cohort (*p* = 0.03) (Table 4). Among **34 patients with the Q703K VUS** mutation, findings were consistent: the Turkish cohort had longer initial attacks (5.4 vs. 3 days, *p* = 0.03), higher initial PGA (7.2 vs. 6, *p* = 0.016) and CRP (5 vs. 2.9 mg/dl, *p* < 0.001), and higher last PGA scores (*p* = 0.03), with other parameters showing no significant differences (Supplementary 1).

#### Treatment approaches, doses, and remission status at last visit

Treatment modalities differed significantly between cohorts (*p* < 0.001). None of the Turkish VUS cohort patients were untreated, whereas 59% of the German VUS cohort received no therapy. Colchicine use was more frequent in the German VUS cohort (30% vs. 10%), while Canakinumab (75% vs. 8%) and Anakinra (15% vs. 3%) were higher in the Turkish VUS cohort (Fig. 2a). Dosing regimens also differed significantly between cohorts (*p* < 0.01). At the last visit, 59% of the German VUS cohort were off treatment. Moreover, above-standard dosing was more frequent in the Turkish VUS cohort (50% vs. 1%), whereas standard dosing was more common in the German VUS cohort (30% vs. 17%) (Fig. 2b). Remission status showed significant differences. Complete remission without therapy was observed in 55% of German VUS cohort patients but none of the Turkish VUS cohort. Complete remission on medication was more frequent in the Turkish VUS cohort (75% vs. 44%). Partial remission on medication (8%) and non-remission (17%) occurred only in the Turkish VUS cohort (Fig. 2c). Among 34 patients carrying the Q703K variant, similar patterns were observed. Turkish cohorts showed higher use of Canakinumab (72% vs. 8%) and Anakinra (14% vs. 3%), while 59% of the German cohort remained untreated. Above-standard dosing was more frequent in the Turkish cohort. Complete remission without therapy occurred only in the German cohort (55%), whereas non-remission was observed exclusively in the Turkish cohort (29%). (Supplementary Figs. 1, 2, 3).

**Table 4:** Comparison of phenotypic, and disease activity assessments in Turkish and German cohorts consisting of 39 patients with VUS mutations

Parameter	Turkish Cohort (*n* = 12)	German Cohort (*n* = 27)	*p*-value (two-sided)
Age at Diagnosis (years), median (range)	7,5 (2–18)	3 (1–16)	**0.011**
Disease Onset Age (years), median (range)	3 (0–13)	2 (1–12)	0,172
Time to Diagnosis (years), median (range)	4 (0–13)	1 (0–8)	**0.002**
**Phenotype**			**0.016**
mild-FCAS, n (%)	0 (0)	7 (25)	
moderate-MWS, n (%)	12 (100)	20 (74)	
**Initial and Follow-Up Assessments**			
Initial Attack Length (days), median (range)	5 (2–10)	3 (2–5)	**0.006**
Initial PGA, median (range)	8 (4–10)	6 (3–9)	**0.016**
Initial PPGA, median (range)	7,5 (5–10)	7 (4–9)	0.270
Initial CRP (mg/dl), median (range)	5,2 (1.2–19.2)	2.9 (0.3–18.2)	**0.002**
Last PGA, median (range)	0 (1,5–5)	0 (0–2)	**0.03**
Last PPGA, median (range)	0 (0–5)	0 (0–2)	0.39
Last CRP (mg/dl), median (range)	0.2 (0.02–0.96)	0.12 (0–0.57)	0.33
Attack Frequency Last Year, median (range)	0 (0–12)	0 (0–5)	0.76

Abbreviations: P/LP, Pathogenic/Likely Pathogenic; VUS, Variant of Uncertain Significance; FCAS, Familial Cold Autoinflammatory Syndrome; MWS, Muckle-Wells Syndrome; CINCA, Chronic Infantile Neurological Cutaneous and Articular Syndrome; PGA, Physician Global Assessment; PPGA, Patient/Parent Global Assessment; CRP, C-Reactive protein. Notes: p-values were calculated using Pearson Chi-Square or Mann-Whitney U tests, as appropriate. Ranges indicate minimum and maximum values, with all medians reported as min–max

**Table 5:** Comparison of clinical symptoms in Turkish and German cohorts consisting of 39 patients with VUS mutations

Parameter	Turkish Cohort *n* (%)	German Cohort *n* (%)	*p*-value
Headache	6 (50)	13 (48)	0,915
Urticarial Rash	11 (92)	18 (66)	**0**,**034**
Fatigue	12 (100)	19 (70)	**0**,**069**
Arthralgia	10 (83)	20 (74)	0,52
Arthritis	7 (58)	5 (19)	**0**,**013**
Diarrhea	1(8)	12 (44)	**0**,**027**
Vomiting	4 (33)	3 (11)	0,095
Abdominal Pain	7 (58)	19 (70)	0,462
Aseptic Meningitis	2 (16)	0 (0)	0,060
Frontal Bossing	2 (16)	0 (0)	**0**,**029**
Thoracic Pain	4 (33)	0 (0)	**0**,**002**
Lymphadenopathy	2 (16)	17 (63)	**0**,**008**
Conjunctivitis	6 (50)	9 (33)	0,32
Fever	12 (100)	21 (77)	**0**,**027**
Aphthous Ulcers	2 (16)	21 (77)	**< 0**,**001**
Family History	3 (25)	17 (63)	**0**,**029**
Stress-triggered Attacks	4 (33)	4 (33)	0,186
Infection-triggered Attacks	1 (8)	20 (74)	**< 0**,**001**
Cold-triggered Attacks	3 (25)	3 (25)	**0**,**267**
Seasonality-triggered Attacks	3 (25)	3 (25)	**0**,**267**

Abbreviations and Notes: p-value: Pearson Chi-Square test for categorical variables. Percentages are rounded to one decimal place. The Total column refers to the sum of participants from Turkey and Germany (*n* = 51)

**Fig. 2a:**
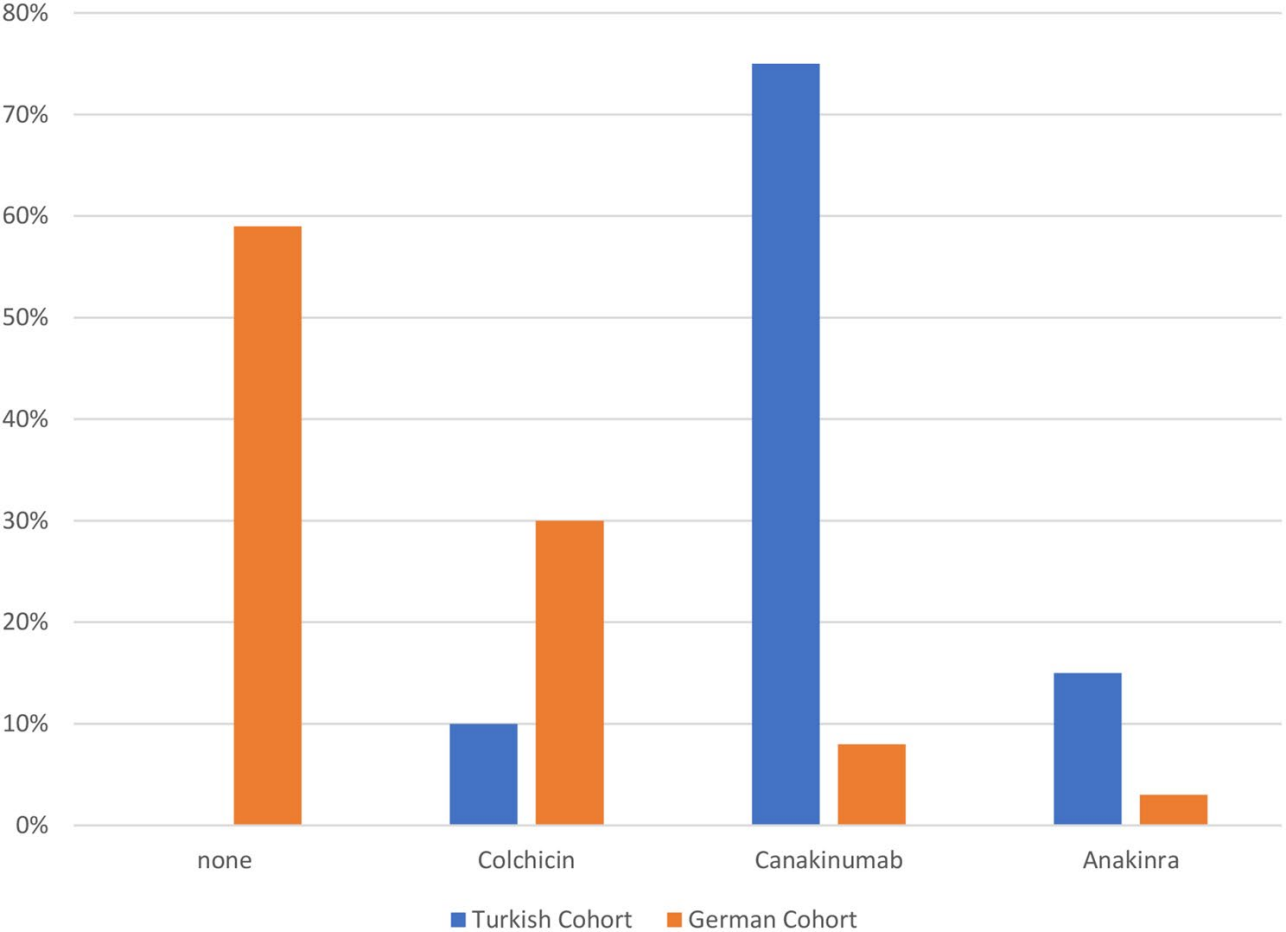
Comparison of last therapies in Turkish and German cohorts consisting of 39 patients with VUS mutations. Legend: In the Turkish cohort, Canakinumab was used in 75%, Colchicine in 10%, and Anakinra in 15%, with no untreated patients. In the German cohort, 59% were untreated, while Canakinumab, Colchicine, and Anakinra were used in 8%, 30%, and 3%, respectively (*p* < 0.001)

**Fig. 2b:**
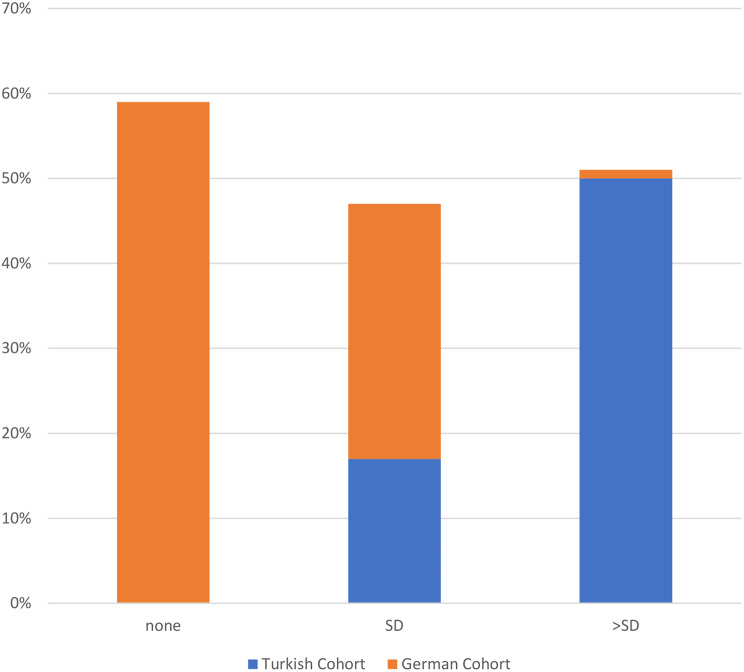
Distribution of last therapy doses in the Turkish and German cohorts Consisting of 39 Patients with VUS Mutations. Legend: Comparison of therapy doses at the last visit in the Turkish (*n* = 12) and German (*n* = 27) cohorts. Doses are categorized as no treatment (none), standard dose (SD) A higher proportion of the Turkish cohort received doses above the standard dose (SD) (50% vs. 1%), while the German cohort had a greater percentage receiving the standard dose (SD) (30% vs. 17%) (*p* < 0.01)

**Fig. 2c:**
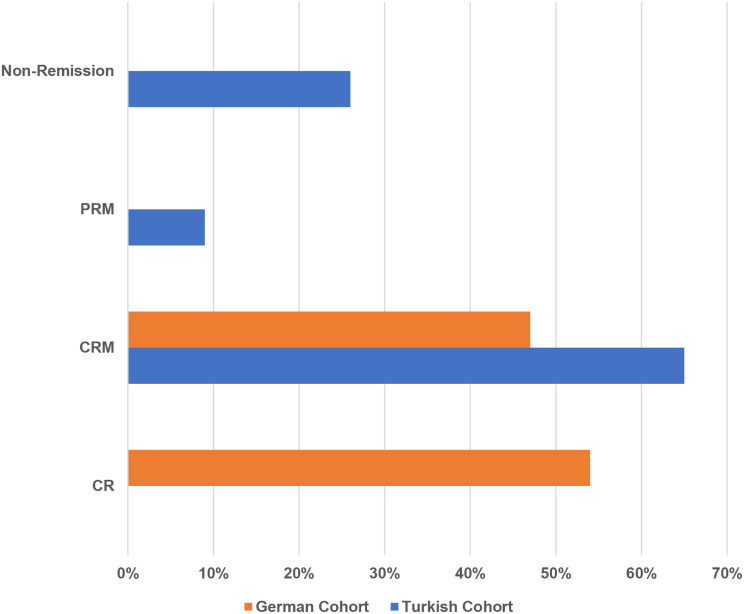
Remission status at last visit by Turkish and German cohorts consisting of 39 patients with VUS mutations. Legend: At the last visit, 75% of the Turkish cohort were in CRM, compared to 44% in the German cohort. In contrast, 55% of the German cohort achieved CR, while none of the Turkish cohort reached this status. Non-remission was observed in 17% of the Turkish cohort, but none in the German cohort. PRM was seen in 8% of the Turkish cohort. Chi-square test results: *p* < 0.001. Abbreviations: CRM: Complete remission with therapy, CR: Complete remission without therapy, PRM: Partial remission with therapy

## Discussion

This study provides the first systematic comparison of Turkish-origin pediatric CAPS cohorts residing in Turkey and Germany, offering novel insights into genotype–phenotype relationships and modifying environmental influences. The Turkish cohort carried a higher proportion of pathogenic or likely pathogenic *NLRP3* variants and demonstrated increased disease activity both at baseline and follow-up, accompanied by greater therapeutic requirements. Remarkably, even within subgroups carrying similar VUS mutations—particularly the Q703K variant—the Turkish cohort exhibited more pronounced systemic involvement and more frequent need for high or above-standard biologic dosing to achieve remission. In contrast, the German cohort displayed overall milder phenotypes, with a substantial proportion maintaining remission without ongoing treatment. These findings suggest that, beyond genetic predisposition, environmental context, healthcare access, and region-specific modifying factors may play a decisive role in shaping disease expression and severity, underscoring the importance of context-aware and individualized management strategies in CAPS.

### A clear genotype–phenotype correlation was evident across both cohorts

 The higher frequency of pathogenic *NLRP3* variants in the Turkish cohort was associated with a more severe disease course, whereas the German cohort—dominated by the Q703K VUS—showed milder phenotypes and instances of treatment-free remission. These findings are consistent with previous reports showing that low-penetrance *NLRP3* variants, such as Q703K or V198M, are often associated with attenuated phenotypes and variable inflammatory activity [10, 14], underscoring the importance of genotype-guided, individualized treatment strategies in CAPS.

### Phenotypic variability in CAPS showed distinct regional differences

 FCAS was observed exclusively in the German cohort, whereas moderate MWS and severe CINCA/NOMID phenotypes were more prevalent in the Turkish cohort. This pattern is consistent with global observations—where FCAS predominates in North America, MWS is most common in Europe, and severe CINCA/NOMID forms are rare and typically arise from de novo variants—supporting the possibility of region-specific influences on CAPS phenotypic presentation [16]. But the absence of FCAS in the Turkish cohort may reflect diagnostic under-recognition rather than a true epidemiological difference, as individuals with cold-induced urticarial rashes are often managed as allergic or idiopathic urticaria rather than being referred for autoinflammatory evaluation.

### Regional variation was also evident in disease triggers and symptom patterns

Infections were the most frequent precipitant in the German cohort, whereas cold exposure and seasonal changes were more commonly reported in Turkey. This suggests that relative temperature fluctuations and host susceptibility, rather than absolute climate, drive flare occurrence. Gastrointestinal manifestations, such as diarrhea and aphthous ulcers, were more frequent in the German cohort—consistent with reports linking Q703K variants to increased gastrointestinal involvement [10]—which may also reflect underlying differences in gut microbiota composition or dietary patterns.

### Environmental factors appeared to influence CAPS expression

As Turkish and German cohorts carrying similar VUS variants—including Q703K—showed divergent disease severity, with the Turkish cohort requiring more intensive biologic therapy to achieve remission. Similar patterns have been reported in other monogenic autoinflammatory diseases. Studies in FMF have shown that environmental and socioeconomic conditions can profoundly modify clinical outcomes. In a large multinational FMF cohort, Touitou et al. identified country of residence as the strongest predictor of renal amyloidosis—surpassing even genetic risk factors [17]. Likewise, two studies by Ozen et al. comparing Turkish FMF cohorts residing in Turkey and Germany demonstrated more severe disease among the Turkish cohort, implicating environmental and healthcare-related disparities as disease modifiers [18, 19]. Given the shared mechanisms of innate immune dysregulation between FMF and CAPS, it is plausible that region-specific influences—such as climate, infection burden, nutritional patterns, and healthcare access—similarly shape CAPS severity and clinical trajectory. These findings suggest that environmental factors may influence the clinical presentation and management of monogenic disease cohorts.

### Diagnostic delays and differences in healthcare awareness may critically influence disease course

As indicated by the longer time to diagnosis and higher baseline inflammatory indices observed in the Turkish cohort. Delayed diagnosis likely contributes to increased disease activity at presentation, a greater need for intensive therapy, and a higher risk of long-term complications. In contrast, the shorter diagnostic intervals in Germany may have facilitated earlier intervention, potentially accounting for lower disease activity and higher rates of treatment discontinuation. When considered alongside elevated initial disease activity markers and longer attack durations, these findings underscore the importance of timely diagnosis and intervention in preventing disease progression and determining long-term outcomes. These observations align with previous studies demonstrating that delayed recognition of CAPS and other monogenic autoinflammatory diseases is associated with more severe clinical manifestations and increased complication rates [1, 16].

Despite the strengths of this bi-national, multicenter study, several limitations should be acknowledged. Although the sample size is notable given the rarity of CAPS, it remains relatively small, potentially restricting the generalizability of the findings. The retrospective design introduces the possibility of recall and selection biases. Moreover, variations in medical record-keeping practices, data availability, and healthcare system structures across Turkey and Germany may complicate direct comparisons of specific clinical parameters. In addition, functional validation studies were not performed for the variants of uncertain significance, limiting the interpretation of their potential pathogenicity. Although all patients underwent a comprehensive autoinflammatory gene panel covering NLRP3, MEFV, MVK, NLRC4, and other relevant genes, rare or novel variants not included in the panel cannot be completely excluded. As most patients carried low-penetrance NLRP3 variants, which are less strongly associated with CAPS, the relative contribution of non-genetic factors to the phenotype may be overestimated in this cohort. Therefore, these findings are not directly generalizable to patients with clearly pathogenic genotypes. In addition, the inclusion of patients based on both diagnostic and classification criteria may have introduced further heterogeneity, as less stringent diagnostic criteria may capture milder phenotypes, thereby influencing the observed cohort differences.

## Conclusion

CAPS is not solely a genetic disorder; it represents a complex, multifactorial condition in which genotype and environmental context converge to shape disease severity and clinical course. These findings underscore the need for personalized, context-aware management strategies that integrate genetic insights with environmental and healthcare considerations. Future international collaborations and registry-based studies will be essential to optimize care, refine diagnosis, and improve long-term outcomes for CAPS patients across diverse settings.

## Supplementary Information

Below is the link to the electronic supplementary material.

Supplementary Material 1

## Data Availability

Source data are available from the corresponding author upon reasonable request.

## Abbreviations

CAPS: Cryopyrin-associated periodic syndromes
CINCA/NOMID: Chronic infantile neurologic cutaneous articular syndrome / neonatal-onset multisystem inflammatory disease
CRP: C-reactive protein
FCAS: Familial cold autoinflammatory syndrome
FMF: Familial mediterranean fever
IL-1β: Interleukin-1 beta
MWS: Muckle–wells syndrome
myAIDAI: Modified autoinflammatory disease activity index
PGA: Physician global assessment
PPGA: Patient/parent global assessment
SD: Standard dose
SAA: Serum amyloid A
VAS: Visual analog scale
VUS: Variants of uncertain significance
