# Supplementary material for: Genotype–phenotype correlations in pediatric CAPS with predominantly low-penetrance NLRP3 variants among Turkish patients in Germany and Turkey: beyond borders
Source: Pediatr Rheumatol Online J. 2026 Apr 11;24:21. doi: 10.1186/s12969-026-01214-7 (PMC13072506; doi:10.1186/s12969-026-01214-7)
Supplement: Supplementary file 1 — Supplementary Material 1 [file 12969_2026_1214_MOESM1_ESM.docx]

**Supplementary Table 1. Comparison of Phenotypic, and Disease Activity Assessments Between the Turkish and German Cohorts with Q703K VUS Mutations**

| **Parameter** | **Turkish**  **Cohort**  **(n=7)** | **German**  **Cohort**  **(n= 27)** | **p-value (two-sided)** |
| --- | --- | --- | --- |
| Age at Diagnosis (years), median (range) | 8.7 (4–18) | 3 (1–16) | **0.02** |
| Disease Onset Age (years), median (range) | 2.7 (0–5) | 2 (1–12) | 0,531 |
| Time to Diagnosis (years), median (range) | 6 (0–13) | 1 (0–8) | **0.003** |
| **Phenotype** |  |  | **0.05** |
| mild-FCAS, n (%) | 0 (0) | 7 (25) |  |
| moderate-MWS, n (%) | 7 (100) | 20 (74) |  |
| **Initial and Follow-Up Assessments** |  |  |  |
| Initial Attack Length (days), median (range) | 5.4 (2–12) | 3 (2–5) | **0.035** |
| Initial PGA, median (range) | 7.2 (4–9) | 6 (3–9) | **0.016** |
| Initial PPGA, median (range) | 7,5 (5–10) | 7 (4–9) | 0.270 |
| Initial CRP (mg/dl), median (range) | 5 (0.3–23) | 2.9 (0.3–18.2) | **<0.01** |
| Last PGA, median (range) | 1.8 (0–5) | 0 (0–2) | **0.031** |
| Last PPGA, median (range) | 1 (0–5) | 0 (0–2) | 0.647 |
| Last CRP (mg/dl), median (range) | 0.3 (0.02–0.96) | 0.12 (0–0.57) | 0.214 |
| Attack Frequency Last Year, median (range) | 2.5 (0–12) | 0 (0–5) | 0.708 |
| Abbreviations: P/LP, Pathogenic/Likely Pathogenic; VUS, Variant of Uncertain Significance; FCAS, Familial Cold Autoinflammatory Syndrome; MWS, Muckle-Wells Syndrome; CINCA, Chronic Infantile Neurological Cutaneous and Articular Syndrome; PGA, Physician Global Assessment; PPGA, Patient/Parent Global Assessment; CRP, C-Reactive protein. Notes: p-values were calculated using Pearson Chi-Square or Mann-Whitney U tests, as appropriate. Ranges indicate minimum and maximum values, with all medians reported as min–max. | | | |

**Supplementary Table 2. Comparison of Clinical Symptoms Between the Turkish and German Cohorts with Q703K Mutations**

| **Parameter** | **Turkish**  **Cohort**  **n (%)** | **German**  **Cohort**  **n (%)** | **p-value** |
| --- | --- | --- | --- |
| Headache | 4 (57) | 13 (48) | 0,671 |
| Urticarial Rash | 7 (100) | 18 (66) | **0,026** |
| Fatigue | 7 (100) | 19 (70) | **0,038** |
| Arthralgia | 7 (100) | 20 (74) | 0,550 |
| Arthritis | 2 (28) | 5 (19) | 0,569 |
| Diarrhea | 1(14) | 12 (44) | 0,122 |
| Vomiting | 2 (28) | 3 (11) | 0,277 |
| Abdominal Pain | 4 (56) | 19 (70) | 0,512 |
| Aseptic meningitis | 0 (0) | 0 (0) |  |
| Frontal Bossing | 1 (14) | 0 (0) | 0,070 |
| Thoracic Pain | 1 (14) | 0 (0) | 0,070 |
| Lymphadenopathy | 0 (0) | 17 (63) | **<0,001** |
| Conjunctivitis | 4 (57) | 9 (33) | 0,25 |
| Fever | 7 (100) | 21 (77) | 0,079 |
| Aphthous Ulcers | 2 (28) | 21 (77) | **0,016** |
| Family History | 1 (14) | 17 (63) | **0,017** |
| Stress-triggered attacks | 2 (28) | 4 (33) | 0,417 |
| Infection-triggered attacks | 0 (0) | 20 (74) | **<0,001** |
| Cold-triggered attacks | 3 (43) | 3 (25) | 0,070 |
| Seasonality-triggered attacks | 3 (43) | 3 (25) | 0,070 |
| Abbreviations and Notes: p-value: Pearson Chi-Square test for categorical variables. Percentages are rounded to one decimal place. The Total column refers to the sum of participants from Turkey and Germany (n=51). | | | |

**Supplementary Figure 1. Comparison of Last Therapies in Turkish and German Cohorts Consisting of 34 Patients with Q703K Mutations**


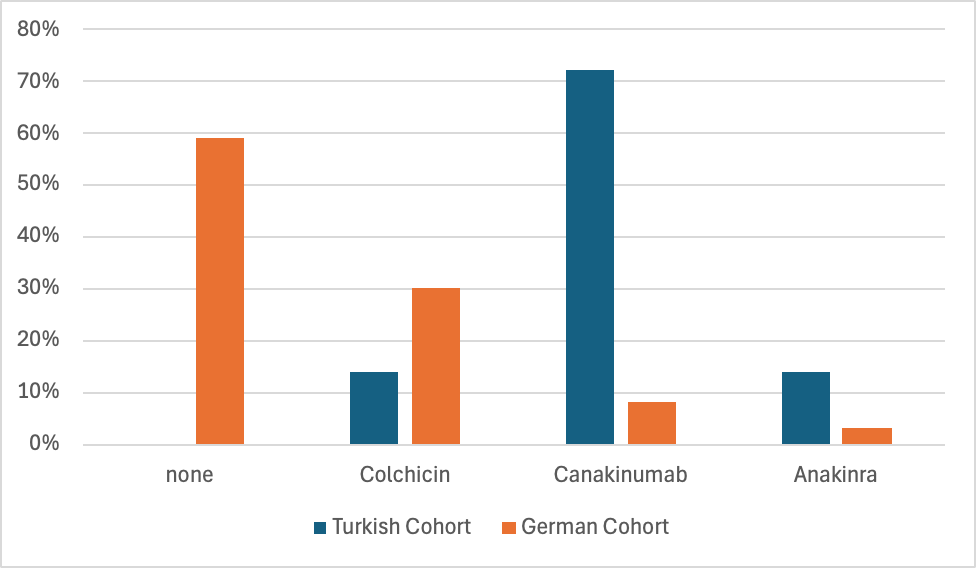


**Legend**: Comparison of Last Therapies in Turkish and German Cohorts Consisting of 34 Patients with Q703K Mutations. The table shows the percentage of patients in each cohort receiving no treatment, Colchicine, Canakinumab, or Anakinra. Differences between cohorts are statistically significant with *p* < 0.01.

**Supplementary Figure 2. Distribution of last therapy doses in the Turkish and German cohorts Consisting of 34 patients with Q703K Mutations**

**Legend:** Distribution of last therapy doses in the Turkish and German cohorts consisting of 34 patients with Q703K mutations. The table shows the percentage of patients receiving no treatment, standard dose (SD), and greater than standard dose (>SD) (p=0.02).

**Supplementary Figure 3. Remission Status at Last Visit in Turkish and German Cohorts Consisting of 34 Patients with Q703K Mutations**


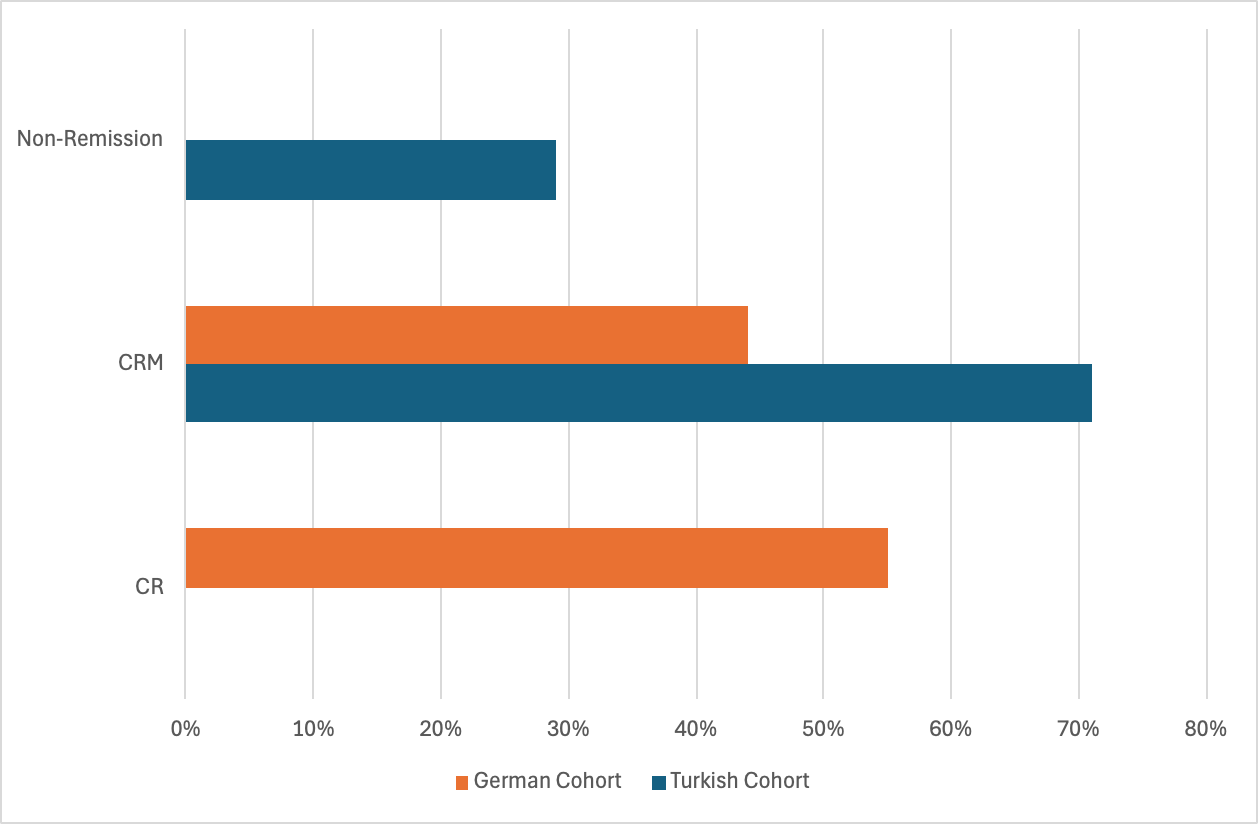


**Legend:** Remission status at last visit in Turkish and German cohorts consisting of 34 patients with Q703K mutations. The table shows the percentages of patients achieving complete remission (CR), clinical remission with medication (CRM), and non-remission. Differences between cohorts are statistically significant (*p* < 0.01).
